# Prediction of Crop Yield Using Phenological Information Extracted from Remote Sensing Vegetation Index

**DOI:** 10.3390/s21041406

**Published:** 2021-02-17

**Authors:** Zhonglin Ji, Yaozhong Pan, Xiufang Zhu, Jinyun Wang, Qiannan Li

**Affiliations:** 1 State Key Laboratory of Remote Sensing Science, Jointly Sponsored by Beijing Normal University and Institute of Remote Sensing and Digital Earth of Chinese Academy of Sciences, Beijing 100875, China; jizhonglin@mail.bnu.edu.cn (Z.J.); zhuxiufang@bnu.edu.cn (X.Z.); 201831051030@mail.bnu.edu.cn (J.W.); lqn@mail.bnu.edu.cn (Q.L.); 2 Institute of Remote Sensing Science and Engineering, Faculty of Geographical Sciences, Beijing Normal University, Beijing 100875, China; 3 Academy of Plateau Science and Sustainability, Qinghai Normal University, Xining 810016, China

**Keywords:** yield prediction, corn, MODIS, NDVI time series, crop phenology, growth phase length, growth rate

## Abstract

Phenology is an indicator of crop growth conditions, and is correlated with crop yields. In this study, a phenological approach based on a remote sensing vegetation index was explored to predict the yield in 314 counties within the US Corn Belt, divided into semi-arid and non-semi-arid regions. The Moderate Resolution Imaging Spectroradiometer (MODIS) data product MOD09Q1 was used to calculate the normalized difference vegetation index (NDVI) time series. According to the NDVI time series, we divided the corn growing season into four growth phases, calculated phenological information metrics (duration and rate) for each growth phase, and obtained the maximum correlation NDVI (Max-R^2^). Duration and rate represent crop growth days and rate, respectively. Max-R^2^ is the NDVI value with the most significant correlation with corn yield in the NDVI time series. We built three groups of yield regression models, including univariate models using phenological metrics and Max-R^2^, and multivariate models using phenological metrics, and multivariate models using phenological metrics combined with Max-R^2^ in the whole, semi-arid, and non-semi-arid regions, respectively, and compared the performance of these models. The results show that most phenological metrics had a statistically significant (*p* < 0.05) relationship with corn yield (maximum R^2^ = 0.44). Models established with phenological metrics realized yield prediction before harvest in the three regions with R^2^ = 0.64, 0.67, and 0.72. Compared with the univariate Max-R^2^ models, the accuracy of models built with Max-R^2^ and phenology metrics improved. Thus, the phenology metrics obtained from MODIS-NDVI accurately reflect the corn characteristics and can be used for large-scale yield prediction. Overall, this study showed that phenology metrics derived from remote sensing vegetation indexes could be used as crop yield prediction variables and provide a reference for data organization and yield prediction with physical crop significance.

## 1. Introduction

Timely and accurate predictions of crop yield before harvest at a large scale is critical for food security and administrative planning, especially in the current continually changing global environment and international situation [1]. At the same time, early-season crop yield predictions are also often required as essential information for decision making in the harvest, processing, storage, transportation, and marketing of agricultural commodities [2].

After decades of research, crop yield prediction methods can be summarized in two groups, i.e., empirical models and process-based models [3]. Empirical models determine the relationship between the prediction parameters and yield and process-based models simulate the growth process of the crop. The latter requires many calibration parameters, which are relatively difficult to obtain. Empirical models are more commonly used in large-scale yield prediction and mainly employed two parameters: environmental and remote sensing variables [4]. The former includes the four most important variables: soil productivity, accessibility of water, climate, and pests or diseases [5]. The rapid development of remote sensing technology has produced more remote sensing variables to serve crop yield prediction, which can be further divided into two types: a variable to monitor crop growth, such as vegetation indices (VIs) and photosynthetic activities [6,7,8,9], and the other variable to describe living conditions, such as heat stress [10,11] and water stress [12,13].

Recently, some studies have employed remote sensing derived phenological variables to predict crop yields [14,15]. These phenological variables are phenological period date information and belong to the variables that monitor crop growth. The date information assesses whether every phenological stage occurs during a period of favorable weather conditions [16] and how an accelerated or delayed phenological stage will affect crop growth conditions, especially when the current climate changes drastically [17]. For example, dates of anthesis, lengths of vegetative and reproductive growth periods, and the growing season can reflect climate change influences [18]. The plant breeding community also has a keen interest in developing crops that “stay-green” for longer, increasing the duration of grain-fill and decreasing senescence rate [19,20,21].

The phenological period date information provides practical support for the development of remote sensing in crop yield prediction. Many models predict crop yield based on remote sensing variables within a fixed timescale [3,4,22,23,24], such as the month. The representation of crop phenological date information is simplified in these models, such that understanding the yield variations is critical because crop growth characteristics and sensitivities toward different environmental events vary with changes in the growth phases (GPs) defined by phenological dates. This condition leads to spatial-temporal heterogeneity between the yield prediction variables. Experiments have shown that phenological dynamic information can solve this heterogeneity issue and improve the yield prediction or estimation accuracy [25,26]. For example, the accumulative leaf area index (LAI) in a specific GP had the highest correlation with the regional crop yield [27], and the time series index, combined with phenological date information, can effectively improve the yield prediction accuracy [28,29].

In addition to the phenological date, one piece of essential phenological information is GP duration [30]. Bai et al. [31] noted that the phase duration could be combined with remote-sensing-based parameters to improve crop yield prediction. The GPs in their study were divided by the effective accumulated temperature. Other than the effective accumulated temperature, the Normalized Difference Vegetation Index (NDVI) is widely used for monitoring crop growth conditions and is an effective way to extract phenology [32,33,34,35]. Magney et al. [36] used ground-based sensors to collect NDVI readings to divide GPs and calculate crop phenological information (the NDVI rate at different GPs and the duration of different GPs) at the field level; the results of their study indicated that NDVI rate and GP duration were good predictors for crop yield. NDVI rate represents the crop growth rate. The crop growth conditions can be reflected under the comprehensive effect of the external environment and crop characteristics by combining the growth duration and rate. Field observation data is a first-hand source of accurate and reliable information for crop phenology research. However, field observation experiments usually require substantial manpower, financial resources, material resources, and time. Therefore, field observations are not suitable as a method to obtain data for long-term and large-scale crop phenology.

Satellite remote sensing technology can effectively obtain long-term and large-scale phenological information. Although the technology has certain limitations, such as surface information accuracy (i.e., mixed pixel) and inherent complexity (i.e., cloud contamination and atmospheric variability), it lowers the cost of large-scale crop monitoring and possesses substantial potential for detecting crop regional phenology patterns through the VI time series [25,37,38]. For example, most remote-sensing-based studies have employed the data from the National Aeronautics and Space Administration’s (NASA) Moderate Resolution Imaging Spectroradiometer (MODIS) [39,40,41]. The spatial resolutions (250-m, 500-m, and 1000-m) are suitable for monitoring different scales from the county-level to the global scale, and the temporal resolutions (8- and 16-day) allow for continuous and in near-real-time monitoring within the whole growing season. Thus, the satellite remote sensing data is suitable to derive phenological metrics (duration and rate). It is also worth investigating the further application of phenological metrics in predicting yield at a large-scale.

The overall goal of this study was to predict corn yield using phenological information metrics extracted from the MODIS-NDVI time series. The specific objectives were to: (i) analyze the relationship between phenological metrics derived from satellite remote sensing VI and the yield, (ii) evaluate the capacity of phenological metrics to predict large-scale corn yield, and (iii) test the ability of the combined phenological metrics and other parameters derived from remote sensing for the prediction of corn yields.

## 2. Materials and Methods

### 2.1. Study Region

**Figure 1:**
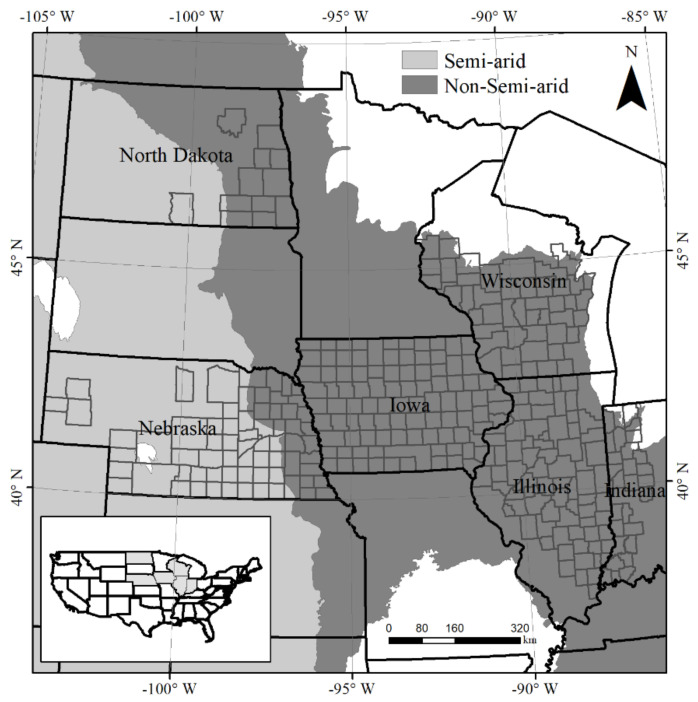
Spatial distribution of selected counties, which are divided into semi-arid and non-semi-arid regions.

The study focused on agricultural counties in six states within the central US Corn Belt, including Illinois, Indiana, Iowa, Nebraska, Wisconsin, and North Dakota. There are a total of 314 counties in which the corn area exceeds 10,000 ha [28], and the mean field size in the US is 19.3 ha [42]. To account for the impact of geographical conditions on crop phenological metrics, the central US Corn Belt was divided into semi-arid and non-semi-arid regions according to the geographic variation in climate, topography, and edaphic conditions (Figure 1).

### 2.2. Data

MODIS 250-m and 8-day composite reflectance product data (MOD09Q1, version 6) for 2008–2018 were acquired from the National Aeronautics and Space Administration (NASA) Reverb (http://reverb.echo.nasa.gov/ (accessed on December 29, 2019)). There were 46 reflectance composites each year. Three MODIS tiles (h10v04, h11v04, and h11v05) were used to cover all counties fully and were re-projected using the MODIS re-projection tool (MRT) to the UTM (Universal Transverse Mercator) system. The 250-m and 8-day reflectance product allows for the calculation of VIs with a higher temporal resolution than that of the standard VI product (MOD13Q1) at 250-m and 16-day.

The county-level corn yields from 2008 to 2018 were obtained from the United States Department of Agriculture (USDA) National Agricultural Statistics Service (NASS) (https://quickstats.nass.usda.gov/ (accessed on 21 April 2019)). The yield estimation unit was converted from bushels acre^−1^ to kg ha^−1^. As some counties lack individual annual yield data, the total number of yield samples was n = 3,320 for the whole region, 460 for the semi-arid region, and 2,860 for the non-semi-arid region. The corn-planting map data were extracted from the 30-m resolution Cropland Data Layer (CDL, http://nassgeodata.gmu.edu/CropScape/ (accessed on 21 April 2019)) from 2008 to 2018, re-projected to match the geographic projection of the MODIS data, and finally used to distinguish pixels dominated by corn from those dominated by other land cover types.

### 2.3. Yield Modeling Approach

**Figure 2:**
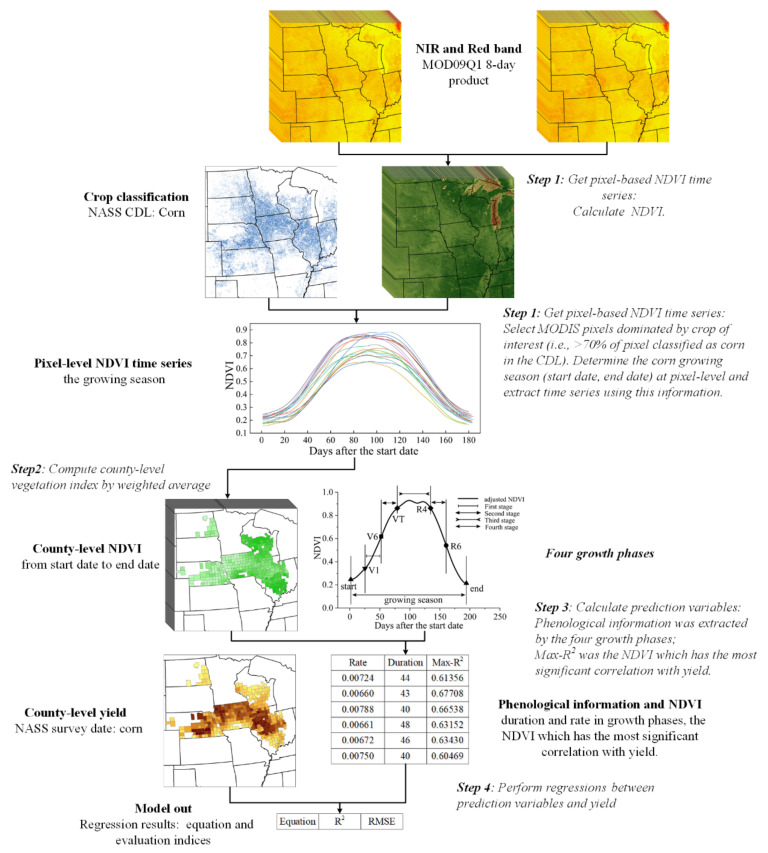
Flow diagram of the datasets and processing used in the model, indicating four steps of model development: 1—obtaining the normalized difference vegetation index (NDVI) time series from the start date to end date of corn at a pixel level, 2—computing the county-level NDVI time series, 3—deriving the prediction variables (duration and rate in four growth phases, maximum correlation NDVI), and 4—constructing the regression relationships between the corn yield and predictors.

Our general approach includes four main steps (Figure 2):

(1) Acquire pixel-based NDVI time series

We used band1 (red, 620–670 nm) and band2 (near-infrared, 841–876 nm) from MOD09Q1 to calculate the NDVI. The MODIS data were processed by an 8-day maximum value composite (MVC), which is less sensitive to clouds and other outliers. However, there are still many random factors that render the NDVI time series data irregular [43]. Thus, the NDVI time series data must be further smoothed to reduce the effects of noise and missing values before extracting the phenological crop characteristics. Popular smoothing methods include the Savitzky–Golay (SG), Double-logistic, and Whittaker Smoother. Previous studies suggested that the SG algorithm can better characterize the temporal signals of corn [44,45]; therefore, we used the SG filter to generate a smooth time series of NDVI on a pixel-by-pixel basis.

Many methods have been proposed to process MODIS data to improve the yield prediction or estimation accuracy [32,41,46,47,48]. The crop spatial distribution map is a vital element of the total crop production, and the ideal approach would be to use it as crop specific masks [32,47]. Mkhabela et al. [41] applied a crop land cover mask to satellite data to remove the effect of non-agricultural land on the NDVI signals, which improved the accuracy of crop yield prediction. We selected the pixels that were dominated by corn (i.e., corn planting area accounts for more than 70% of the MODIS-NDVI pixel area) as the corn pixels. The percentage of corn planted area in the pixels in each year was calculated using the corn planting map of the corresponding year.

**Figure 3:**
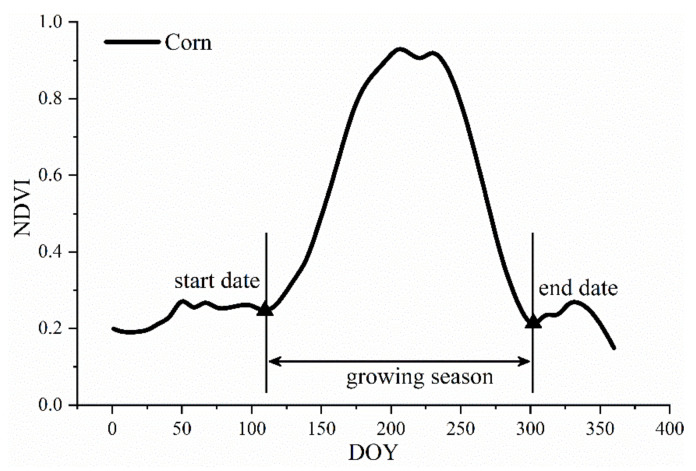
Schematic on how to determine the growing season at the pixel level. NDVI values were extracted from the start date of the growing season to the end date of the growing season for each corn pixel. The “phenologically adjusted” time series values of each corn pixel started with the start date. DOY denotes Day of Year.

In addition, crop planting dates and phenology vary with the location and external environment in every year. Thus, using a fixed calendar date in time series data to build remote-sensing-based yield prediction models is not optimal. A previous study showed that using the green-up date to adjust the start of the VI time series based on pixels can improve the remotely sensed yield prediction of both intra- and inter-annual variability in corn and soybeans [28]. Therefore, in this study, we defined the “phenologically adjusted” NDVI time series pixel by pixel and year by year (Figure 3). We first derived the daily NDVI for each corn pixel based on the 8-day NDVI data using cubic spline interpolation. Then, we defined the date when the NDVI curve began to increase at the bottom of the valley before the single NDVI corn peak as the start date (SD) of the corn growing season. The date when the NDVI reached the bottom of the valley after the single NDVI corn peak was defined as the end date (ED) of the corn growing season. The second derivatives of NDVI at SD and ED were approximately zero. Pixels before SD and after ED were excluded from the analysis, and the time series was adjusted based on SD. Thus, we created “phenologically adjusted” time series values for NDVI per corn pixel.

(2) Compute county-level NDVI time series

As the corn yield was recorded at the county level, we aggregated the daily NDVI of corn pixels in each county to obtain the daily county-level NDVI. To do this, the selected corn pixels were weighted by their contribution, which was the proportion of corn planting area in each pixel. Then, the NDVI values for each county were calculated by a weighted average of these pixels and weights.

(3) Calculation of the prediction variables

Three types of predictors (two phenological metrics [1,2] and one NDVI parameter [3]) were calculated using the county-level NDVI time series (step 2) and used as input variables to predict the corn yield:

Ref. [1] Duration (Equation (1)): Growth duration refers to the number of days in a given crop GP and is calculated by the day of year (DOY) of the end of the GP minus the DOY of its start.

(1) $$Duration\;=\;GP_{end}-GP_{start},$$

where Duration  is the phenological metric of duration; $GP_{end}$ is the DOY of the end of the GP; and $GP_{start}$ is the DOY of the start of the GP.

Ref. [2] Rate (Equation (2)): The rate (slope) of NDVI in a given GP refers to the change rate in the NDVI values throughout the GP.

(2) $$Rate=\frac{NDVI_{GP\left(end\right)}-NDVI_{GP\left(start\right)}}{Duration},$$

where Rate is the phenological metric of rate; $NDVI_{GP\left(end\right)}$ is the NDVI value at $GP_{end}$; $NDVI_{GP\left(start\right)}$ is the NDVI value at $GP_{start}$; and Duration  is the phenological metric of duration.

Ref. [3] Maximum correlation NDVI (Max-R^2^): The Max-R^2^ [29] is the original NDVI value that has the most significant correlation with corn yield in the NDVI time series. The NDVI time series used to extract Max-R^2^ started with the SD.

**Figure 4:**
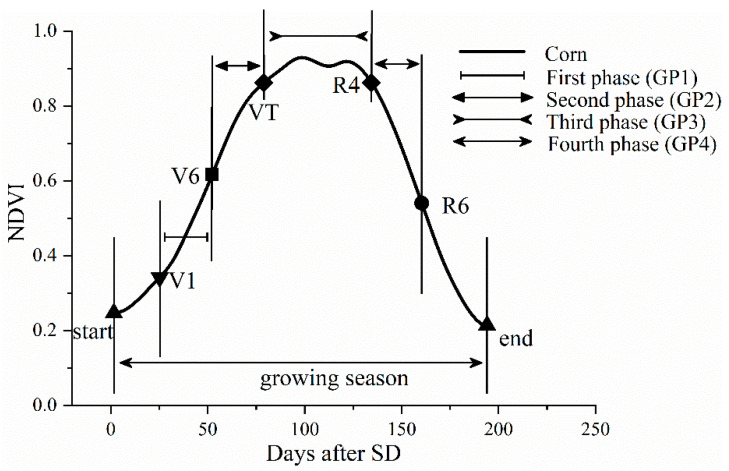
Schematic of the four growth phases for the county-level corn NDVI time series values of the growing season. SD denotes the start date of the corn-growing season.

To extract the above two phenological metrics, four corn GPs were examined (Figure 4): the first phase (GP1) was from V1 to V6, the second phase (GP2) was from V6 to VT; the third phase (GP3) was from VT to R4, and the fourth phase (GP4) was from R4 to R6. The following V1, V6, VT, R4, and R6 refer to the start dates of the emergence, jointing, tasseling, dough, and maturity stages, respectively. The dates of V1, VT, and R4 were extracted using the dynamic threshold method [49]. During the rising phase of the daily corn NDVI time series curve, the points in time where the values increased by a certain value were defined as the date for V1 and VT. Setting to 10% of the distance between the minimum (value at SD) and the maximum is V1 and 90% of the distance between the minimum and maximum is VT, above the minimum. The date of R4 was defined from the descending phase of the corn NDVI time series curve as the point in time at which the value increased by a certain value, currently set to 10% of the distance between the maximum and minimum (value at ED), below the maximum. The date of V6 was defined as when the curvature reaches its local maximum value in the rising curve, where the stalk grows rapidly. The date of R6 occurred in the middle of the senescence phase [39] and was defined as when the curvature reaches its local maximum value in the descending curve. To obtain V6 and R6, the NDVI time series at the county level was fit by a piecewise logistic function [38], resulting in two functions for the rising and descending curves. Then, taking the derivation of the logistic functions, the maximum values of the two derivative functions were denoted as V6 and R6, respectively.

The first growth phase, second growth phase, third growth phase, and fourth growth phase were abbreviated as GP1, GP2, GP3, and GP4, respectively. To facilitate the description of these phenological metrics predictor variables, we defined GP1 duration, GP1 rate, GP2 duration, GP2 rate, GP3 duration, GP3 rate, GP4 duration, and GP4 rate as GP1D, GP1R, GP2D, GP2R, GP3D, GP3R, GP4D, and GP4R, respectively.

(4) Yield regression model

We built three groups of yield regression models for three regions: whole (semi-arid and non-semi-arid), semi-arid, and non-semi-arid. In the first group, we constructed univariate yield regression models for each predictor variable calculated in step 3 using different functions (linear, quadratic, logarithmic, etc.), by which we evaluated the relationship between each predictor variable and corn yield. In the second group, we constructed multivariate yield regression models using phenological metrics and assessed the performance of phenological metrics with respect to the yield prediction. In the third group, we constructed multivariate yield regression models using phenological metrics combined with Max-R^2^ to evaluate the capability of combining phenological metrics with other types of NDVI remote sensing parameters for yield prediction. Both the second and third group models were built using a stepwise regression method, which can select significant variables into the regression equation and reduce collinearity. The standardized regression coefficients in the regression equation were used to compare the importance of different predictor variables on the dependent variable (corn yield).

### 2.4. Model Evaluation

To evaluate the performances of the prediction models in the second group, we used leave-one-year-out cross-validation [3], in which the model was iteratively trained on 10 years of data and then used to predict yield in the held-out year [28] from 2008 to 2018. The metrics used were the coefficient of determination (R^2^) and the root-mean-square error (RMSE). The R^2^ was the predictive model R^2^, and the RMSE was calculated between the actual and predicted yields. For the models in the first and third groups, we only used R^2^ to evaluate the performances. We also used the variance inflation factor (VIF) (Equation (3)) to measure collinearity for the variables in the regression prediction model. In general, a VIF value of less than four indicates non-collinearity [50].

(3) $$VIF_{i}=\frac{1}{1-R_{i}^{2}}$$

where $R_{i}$ is multiple correlation coefficient between the *i*-th variable, $X_{i}$, and all other variables, $X_{j}\;\left(j=1,\;2,\ldots ,k;j\neq i\right)$, and the multiple correlation coefficient is the arithmetic square root of the coefficient of determination $R^{2}$.

## 3. Results

### 3.1. MODIS-Derived Phenological Dates

**Figure 5:**
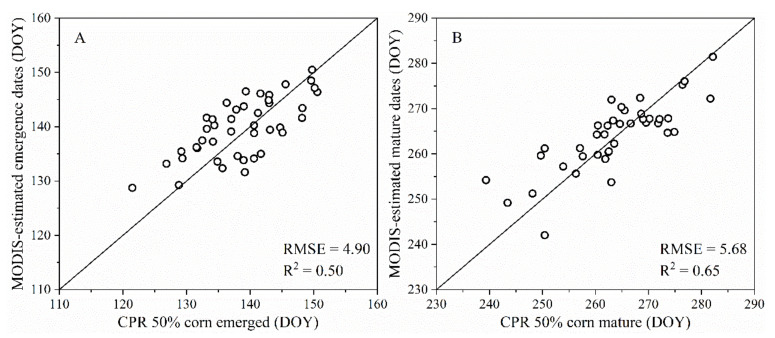
Comparison of the Moderate Resolution Imaging Spectroradiometer (MODIS)-derived emergence date (V1) values, mature date (R6) values, and the United States Department of Agriculture (USDA) Crop Progress Reports (CPR) survey data of 50% corn emerged, mature dates at the state level: (**A**) V1 estimation; (**B**) R6 estimation.

MODIS-derived corn emergence and mature values were compared with the 50% corn emerged and mature dates from Crop Progress Reports (CPR) (2008–2018) (Figure 5) at the state level. The county sample numbers in Indiana and North Dakota were small, and these counties only covered a small part of the state’s spatial range. Therefore, we only compared the results of Illinois, Iowa, Nebraska, and Wisconsin. The R^2^ was 0.50 and 0.65 for the corn emerged and mature, respectively. The corresponding RMSE values were 4.90 and 0.65 days, respectively. The results fell neatly around the 1:1 line.

### 3.2. Relationship between Predictor Variables and Yield

For each predictor variable, we established a set of univariate regression models with different functions, such as linear, quadratic, and logarithmic, and obtained the R^2^ of each model. The largest R^2^ values in the multiple univariate models are listed in Table 1. Most phenological metrics had a statistically significant relationship with the yield (at the *p* < 0.05 level) in the whole, semi-arid, and non-semi-arid regions, except for the GP2 duration and the GP3 rate. The GP1 rate, GP2 rate, GP3 duration, and GP4 rate could be used as yield prediction parameters with relatively large R^2^ values (>0.20).

**Figure 6:**
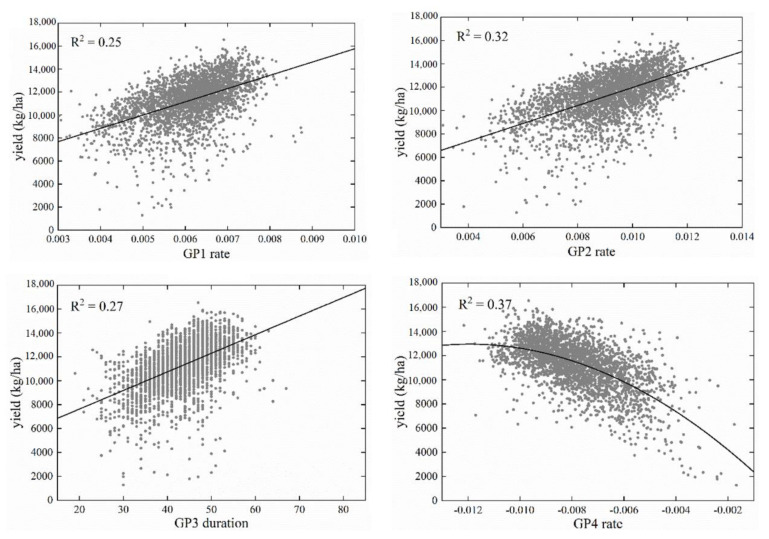
Relationship between yield and GP1 rate, GP2 rate, GP3 duration, and GP4 rate in the whole region. The four phenological predictor variables have the best explanatory power (R^2^ > 0.20) for corn yield among the eight variables.

Increasing the growth rate in GP1 and GP2, extending the growth duration in GP3, and increasing the senescence rate in GP4 are beneficial for increasing the yield (Figure 6). GP1 is in the early stage within the whole growing season, where faster growth is better for the corn. GP2 includes the jointing stage, which is important for crop growth, and the interpretation power of it is stronger than GP1. GP3 is at growth peak; extending the time that the crop remains green helps the crop accumulate more nutrients. Increasing the senescence rate in GP4 ensures that more nutrients are transferred to the grain within a certain time. In addition, the R^2^ values of all phenology metrics were similar in the whole region, non-semi-arid region, and non-semi-arid region, which indicated that the relationship strength between these metrics and corn yield was similar in different regions.

### 3.3. Yield Prediction with Phenological Metrics

The prediction models built with phenological metrics obtained the results with R^2^ = 0.64, 0.72, and 0.64 in the whole region, semi-arid region, and non-semi-arid region (Table 2). More than 60% of the yield was explained by the combination of growth duration and rate. The best yield prediction with a maximum R^2^ value (0.72) was in the semi-arid region. Prediction models did not have the problem of multi-collinearity with VIF values for all metrics < 4.

The four most essential metrics were selected by stepwise regression from eight metrics to build the models. For the whole region and non-semi-arid region, the GP2R presented the highest values for the standardized coefficient (0.70–0.74), followed by GP2D (0.52–0.60), GP3D, and GP4R. The GP2 belongs to the vegetative stage when the stems and leaves grow vigorously and continuously accumulate nitrogen. Crops will provide more nutrients to the ear during the reproductive stage with a longer time or a faster rate to store nitrogen in the vegetative stage [51]. GP3 contains the NDVI time series peak, and there is a positive correlation between leaf area duration (LAD) and corn yield during GP3 [19]. The GP4R had the smallest impact on yield among the four most essential metrics. The GP1R, which had a relatively large explanatory ability for the yield in Table 1, was not selected in the models, indicating there was collinearity among all phenological metrics. For the semi-arid region, beside GP2D, GP2R, and GP3D, GP4D was also a critical impact factor, which indicated that in the semi-arid region, the longer the fourth stage, the higher the crop yield.

**Figure 7:**
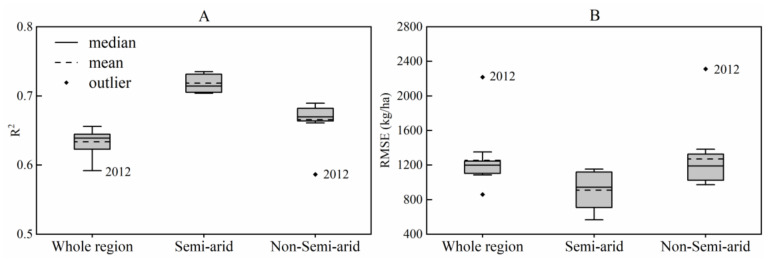
Boxplot of phenological yield prediction model performance for (**A**) R^2^ and (**B**) RMSE in the whole region, semi-arid region, and non-semi-arid region. The models were built with the four most significant phenological metrics using leave-one-year-out cross-validation from 2008 to 2018.

Figure 7 shows the leave-one-year-out cross-validation results of the phenological yield prediction models constructed with the four most significant metrics (Table 2) in the three regions from 2008 to 2018. The medians of the R^2^ values were 0.6–0.8, and the medians of the RMSE values were 900–1200 kg ha^−1^. Results from the semi-arid region presented the highest R^2^ and lowest RMSE, followed by the non-semi-arid region, and the results in the whole region presented the worst R^2^ and RMSE. In addition, the results for 2012 were different from those of other years in the whole and non-semi-arid regions with the lowest R^2^ values (Figure 7A).

### 3.4. Yield Prediction with Phenological Metrics and NDVI

The combination of three phenological metrics variables (GP1D, GP3D, and GP4D or GP1R, GP3D, and GP4R) and Max-R^2^ improved the performance of the Max-R^2^ yield prediction models (Table 1), with a higher R^2^ value of 0.65, 0.73, and 0.68 (Table 3) in the whole region, semi-arid region, and non-semi-arid region, respectively. The prediction models in Table 3 did not have the problem of multi-collinearity with VIF values for all metrics < 4.

After adding the maximum correlation NDVI (Max-R^2^), the phenological metric variables used to construct the multivariate regression model changed compared with phenological metric models (Table 2). For the semi-arid region, the variables changed from the combination of GP2D, GP2R, GP3D, and GP4R to that of GP1R, GP3D, and GP4R. Combining the growth state, time, and rate, the yield prediction model will have more biophysical significance and better results in the semi-arid region. For the non-semi-arid region, the variables changed from the combination of GP2D, GP2R, GP3D, and GP4R to that of GP1D, GP3D, and GP4D. The Max-R^2^ replaced all rate variables indicating that crop growth state and time are more important than the growth rate in a relatively humid environment. The whole region contains more counties located in the non-semi-arid region, and its model variables are the same as that in the non-semi-arid region.

## 4. Discussion

### 4.1. Contributions of This Study

First, this study demonstrated the feasibility of phenological metrics derived from satellite remote sensing data for crop yield prediction. The first group showed that some phenological metrics (durations and rates) have interpretation ability to the corn yields (R^2^ ranged from 0.18 to 0.44 in Table 1) in the three regions, but the ability was limited (maximum R^2^ = 0.44). Compared with this condition, the multivariate regression models built with some phenological metrics in the second group improved the yield prediction accuracy. The multivariate phenological metrics models’ stability and validity were proved through leave-one-year-out cross-validation, and these models can explain 60–80% of the yields. It indicated that phenological metrics from emergence to maturity were meaningful for crops and could be used as input variables to predict yield. Multivariate regression models in the third group built with some phenological metrics and NDVI obtained better yield prediction results than the NDVI univariate regression models in the first group. This indicates that phenological metrics derived from the NDVI time series could be incorporated with other parameters to improve yield prediction in large-scale.

Besides, our result is a useful supplement to phenological variables. Previous studies [14] had proven that phenological variables (phenological date) were closely correlated with crop yields. Phenological date variables can, directly and indirectly, influence the photosynthesis and respiration, which will change the accumulation of effective dry matter. The accumulation of effective dry matter is also affected by the time and rate of photosynthesis and respiration. We used statistic methods to investigate the impacts of phenological metrics (duration and rate) on corn yields. Some phenological metrics can achieve yield prediction, which had the interpretation ability of 60–80% in this paper. We recommend adopting combined phenological date and metrics variables in the future applications related to agricultural yield predictions. Besides, relationships between the phenological metrics derived from MODIS data at a large scale and yield were consistent with the actual growth characteristics of crops in the field. The relationships help the management department make agricultural production decisions in a unified manner. For example, fertilizing before and after the jointing stage increases the growth rate of corn.

Finally, this study provides a method reference for establishing the yield prediction model of other crops. The R^2^ between the yield and NDVI rate of GP1, GP2, and GP4 ranged from 0.27 to 0.44, indicating that it may be more beneficial to organize time series data parameters based on the GP [25] and provide support for dynamic yield predictions with the growth stages as a time unit [26]. The combination of the duration and rate in each GP can simply simulate the crop growth process. Each crop has its growth characteristics at different growth stages, and the characteristics of each growth stage can be described by the growth duration time and rate. Thus, the models constructed with phenological metrics are based on the inherent growth and development of crops, and the yield prediction method is applicable to other crops (e.g., soybean and wheat).

### 4.2. Factors Affecting Model Accuracy

Our proposed yield prediction method may be affected by the following factors. First, the spatial resolution of the NDVI time series has an impact on our method. The models’ explanatory power in the second group was relatively weak compared with the phenological yield prediction models based on the NDVI time series derived from ground-based sensors [36]. This situation is understandable because there is a gap between the county-level NDVI obtained with 250-m resolution pixels and the NDVI obtained from the ground-based sensors. The commonly used MODIS-based 250-m products are suitable for many regions, such as the Great Plains of the US, which have large field sizes (mean field size of 19.3 ha [42]), and countries in Europe [4,47,52], which have small field sizes (two-thirds of Europeans fields are less than 5 ha [53]). Many methods (pixel-based crop planting ratio, phenological information, among others) have been proposed to improve the accuracy of MODIS in agricultural applications [28,41,46,52,54], such as crop map masks and phenological information adjustment used in this study. The NDVI is the most commonly used vegetation index, calculated from the two bands of the MODIS 250-m reflectivity products. Crop-specific NDVI selected by the crop mask [2,46] contains signals from all land surface types; therefore, it is still a mixture of the signals, which partially affects the accuracy of the phenology extraction and yield prediction.

Second, the NDVI time series need be collected from years with different climate condition (such as wet years and dry years). The yield prediction method using phenological metrics works best in the semi-arid region (Table 2, Figure 7). The United States suffered a drought in 2012, resulting in severe crop yield losses [55]. For the whole and non-semi-arid region, the explanatory power (R^2^) for models constructed with data including 2012 (average R^2^ = 0.64, 0.67) was higher than that of models built without 2012 data (R^2^ = 0.59, 0.59). It indicates that phenological metrics can respond to disasters, and datasets containing disaster information can describe more environmental characteristics. Thus, the model constructed using the datasets of 2012 can provide more yield information. Models constructed with data that did not include disaster information had higher RMSEs when predicting the yield in 2012 (Figure 7B—whole region/non-semi-arid region). However, the semi-arid region did not indicate the above situation. Irrigated corn was mainly planted in semi-arid regions [56], and farmers focused more on agricultural water management to alleviate the impacts of the drought.

Third, the determination of the phenology stage dates has potential impacts on our results. We divided the whole corn growing season into four relatively large GPs based on the MODIS-NDVI time series and corn growth characteristics. The phenological dates determine each GP, and the smoothing methods and phenology extraction methods based-on VI time series jointly determine the extraction of phenological dates. Besides, NDVI is sensitive to high-density vegetation and has a saturation phenomenon [57]. The NDVI saturation occurs in the curve peak of time series, which also influences the phenology stage dates and further affects the GP2, GP3, and GP4. Because there are generally two types of peaks, i.e., steep [54] and steady [58], this was consistent with the peaks obtained in this study. The peak’s steepness refers to two phenological dates in the GP3 and one phenological date in the GP2 (GP4) determined by the phenological extraction method. However, the effect of smoothing (phenology extraction) methods and NDVI saturation are relatively minor. The R^2^ was 0.50 and 0.65 for corn emerged and mature, respectively. The corresponding RMSE values were 4.90 and 0.65 days. The phenological metrics models’ interpretation ability is more than 60%. Based on the above-mentioned phenology dates and yield models accuracy, we proved the feasibility of our method and showed that the phenological information metrics obtained from remote sensing data could be used to predict large-scale yield.

### 4.3. Direction of Future Improvement

The ability to predict yield using phenological metrics can be further improved. Further research should attempt to select pixels with a higher crop planting proportion to weaken the effect of sub-pixel mixtures. It also can use other indices, such as the enhanced vegetation index (EVI) and wide dynamic range vegetation index (WDRVI) [25,39], to obtain phenological information to avoid the impact that high-density vegetation cover has on NDVI saturation. Some remote sensing-based indices can also attempt to extract phenological information, such as the solar induced florescence (SIF), thermal decay rate, and vegetation optical depth (VOP), given that they have all become available at higher spatial resolutions. The SIF can capture vegetation’s photosynthesis process, the thermal decay rate monitors vegetation through diurnal temperature variations [59], and the VOP is highly sensitive to the water content and above-ground biomass of vegetation.

We used stepwise regression models to prove that the selected phenological metrics can be used to predict crop yield. Generally speaking, the use of more parameters can more comprehensively describe the crop growth environment conditions and growth status, which is conducive to better prediction of yield. In the future, we can combine phenological metrics with other parameters, such as the climate and vegetation index, as input to machine learning or deep learning which can effectively solve collinearity and extensively investigate the data features to predict the yield. These climate and vegetation index parameters also can take the phenological stage as the time unit, and combining phenology, climate, and vegetation index data to explore the ability to dynamically predict yield in the continuous phenological stages.

## 5. Conclusions

We used the MODIS MOD09Q1 product to calculate the corn NDVI time series and then divided it into four GPs. The phenological information metrics (duration and rate information) obtained in each GP were used to analyze the relationships between them and the corn yield and combine with the maximum correlation NDVI to build yield prediction models.

We obtained two main conclusions from the results of this study. First, most phenological metrics (duration and rate in different phases) extracted from the MODIS-NDVI time series strongly correlate with corn yield. Some phenological metrics can be combined to predict corn yield with relatively good results at a large scale. As a result of the interaction between crops and the environment, phenological information is a comprehensive and indirect crop yield indicator. It can be applied to yield predictions or estimations for other crop types (e.g., soybeans, wheat, cotton, and rice) and other regions. Second, phenological metrics can also be combined with other types of parameters, such as the maximum correlation NDVI, to improve the yield prediction or estimation accuracy.

The yield is the comprehensive performance of crop growth conditions throughout the season. Dividing the season into multiple phases and using duration and rate extracted from NDVI to describe crop growth duration and rate in different phases can simulate the crop growth. Crops require different environmental conditions and have different growth characteristics at different GPs. The NDVI rate based on GP shows a strong relationship with the yield. Therefore, for other yield predictions or estimation parameters, using the GP as the time scale to avoid geospatial data heterogeneity may be more reasonable, as the crop phenology varies by location and changes from one year to the next. A limitation of this study is that it did not clarify the effect of the time series smoothing methods, phenology extraction methods, and GP division method on the establishment of yield models. Moreover, there is no comparison among the performances of the different vegetation indices in this yield prediction method. In the future, these two aspects can be further analyzed to broaden the applicability of the current study to include more crop and vegetation indices’ diversity characteristics and to understand any limitations that may be present in the method.

## Figures and Tables

**Table 1:** R^2^ between the duration (days), rate (∆NDVI/days), and Max-R^2^ and yield (kg/ha). The whole region contains all counties located in the semi-arid and non-semi-arid regions; semi-arid refers to the counties in the semi-arid region and non-semi-arid refers to the counties in the non-semi-arid region.

Phenological Predictor Variable	Whole Region	Semi-Arid Region	Non-Semi-Arid Region
GP1 duration	0.04 *	0.14 *	0.02 *
GP1 rate	0.25 *	0.35 *	0.23 *
GP2 duration	0.18 *	0.40 *	0.19 *
GP2 rate	0.32 *	0.44 *	0.30 *
GP3 duration	0.27 *	0.43 *	0.25 *
GP3 rate	0.01	0.01	0.01
GP4 duration	0.05 *	0.33 *	0.02
GP4 rate	0.37 *	0.35 *	0.38 *
Max-R^2^	0.61 *	0.66 *	0.62 *

* Significant at *p* < 0.05.

**Table 2:** Results from stepwise multiple linear regression between phenological metrics during all phenological phases and yield with data from 2008 to 2018.

Region	Equation	R^2^	VIFs
Whole region	Y = −10,084.25 + 360.58GP2D + 953,476.78GP2R + 97.00GP3D − 102,453.38GP4R ^a^	0.64	X < 3.10
	Y = 0.52GP2D + 0.70GP2R + 0.32GP3D − 0.08GP4R ^b^		
Semi-arid region	Y = −4716.91 + 155.34GP2D + 483,419.05GP2R + 110.04GP3D + 94.97GP4D ^a^	0.72	X < 1.63
	Y = 0.24GP2D + 0.35GP2R + 0.36GP3D + 0.23GP4D ^b^		
Non-Semi-arid region	Y = −13918.97 + 448.02GP2D + 1,084,391.95GP2R + 107.47GP3D − 127,325.54GP4R ^a^	0.67	X < 3.01
	Y = 0.60GP2D + 0.74GP2R + 0.35GP3D − 0.09GP4R ^b^		

^a^: unstandardized; ^b^: standardized.

**Table 3:** Results for stepwise multiple regression between Max-R^2^ with rate and duration and yield. Dates from 2008 to 2018.

Region	Equation	R^2^	VIFs
Whole region	Y = −11,872.08−56.01GP1D + 45.88GP3D + 56.74GP4D + 27,674.64Max-R^2^	0.65	X < 1.60
Semi-arid region	Y = −12,081.43 + 46,3358.80GP1R + 56.88GP3D + 545,886.68GP4R + 28,890.95Max-R^2^	0.73	X < 2.61
Non-Semi-arid region	Y = −15530.44−77.19GP1D + 56.39GP3D + 72.03GP4D + 31,959.49Max-R^2^	0.68	X < 1.46
